# Differential venom gland gene expression analysis of juvenile and adult scorpions *Androctonus crassicauda*

**DOI:** 10.1186/s12864-022-08866-1

**Published:** 2022-09-08

**Authors:** Fatemeh Salabi, Hedieh Jafari

**Affiliations:** Department of Venomous Animals and Anti-Venom Production, Agricultural Research, Education and Extension Organization (AREEO), Razi Vaccine and Serum Research Institute, Ahvaz, Iran

**Keywords:** Scorpion, Venomous, Transcriptomic analyses, *Androctonus crassicauda*, Growth stages

## Abstract

**Background:**

The *Androctonus crassicauda*, belonging to the genus *Androctonus* of the family Buthidae, is the most venomous scorpion in Middle East countries. However, the venom gland transcriptome profile of *A. crassicauda* scorpion has not yet been studied. In this study, we elucidated and compared the venom gland gene expression profiles of adult and juvenile male scorpion *A. crassicauda* using high-throughput transcriptome sequencing. This is the first report of transcriptional analysis of the venom glands of scorpions in different growth stages, with insights into the identification of the key genes during venom gland development.

**Results:**

A total of 209,951 mRNA transcripts were identified from total RNA-seq data, of which 963 transcripts were differentially expressed (DE) in adult and juvenile scorpions (*p* < 0.01). Overall, we identified 558 up-regulated and 405 down-regulated transcripts in the adult compared to the juvenile scorpions, of which 397 and 269 unique unigenes were annotated, respectively. GO and KEGG enrichment analyses indicated that the metabolic, thermogenesis, cytoskeleton, estrogen signaling, GnRH signaling, growth hormone signaling, and melanogenesis pathways were affected by two different growth conditions and the results suggested that the DE genes related to those pathways are important genes associated with scorpion venom gland development, in which they may be important in future studies, including Chs, Elovl, MYH, RDX, ACTN, VCL, PIP5K, PP1C, FGFR, GNAS, EGFR, CREB, CoA, PLCB, CALM, CACNA, PKA and CAMK genes.

**Conclusions:**

These findings broadened our knowledge of the differences between adult and juvenile scorpion venom and opened new perspectives on the application of comparative transcriptome analysis to identify the special key genes.

**Supplementary Information:**

The online version contains supplementary material available at 10.1186/s12864-022-08866-1.

## Background

Scorpions are venomous predatory arthropod animals belonging to the class Arachnida, which have gained more attention from researchers not only because of their venom component but also because of their ancient origin, which are considered living fossils [1, 2]. In previous, scorpion studies focused on morphological and phylogenetic analysis, while for decades, most studies have focused on isolation and biochemical characterization of specific venom components.

Scorpion venom is a complex mixture of biogenic amines, low molecular weight (MW) peptides, high MW proteins, mucoproteins, and other components which are produced by the columnar epithelial cells of the venom gland [3]. The number of unique natural compounds of most animal's venom is highly variable. For instance, the scorpion's venom contained from 300 to over 1,000 unique molecular entities [4] but it has not been fully proven yet. Up until now, the protein components of scorpion venom have been divided into two categories: small peptides (< 10 kDa) and large proteins (> 10 kDa). Small peptides are the most studied scorpion venom components, which mainly exhibit neurotoxic properties, while the large protein derivatives of scorpion venom have shown enzymatic activities [5]. Mainly due to the heterogeneity, complexity, and high variability of venom composition, as well as the low molecular mass compounds present in scorpion's venom, the current number of reported scorpion venom peptides is much less than the expected ones [6–8].

The emergence of advanced techniques, such as high-throughput RNA-seq, promises venom peptides sequence evolution and novel venom component prediction. RNA-Seq or transcriptome sequencing is one of the currently developed methods utilized for novel gene identification, biology, medicine, and molecular phylogenetic analysis, especially in non-model organisms [9]. Nowadays, the use of transcriptomic analysis has become essential in venomics studies, as this analysis may represent a promising lead for discovering new venom components. The first scorpion venom gland cDNA library, which constitutes collections of cDNA sequences cloned into vectors, was generated in 1989 for the scorpion *A. australis* [10]. Thereafter, transcriptomic analyses of scorpion venom glands have been greatly enhanced by the advent of high-throughput sequencing.

**Fig. 1:**
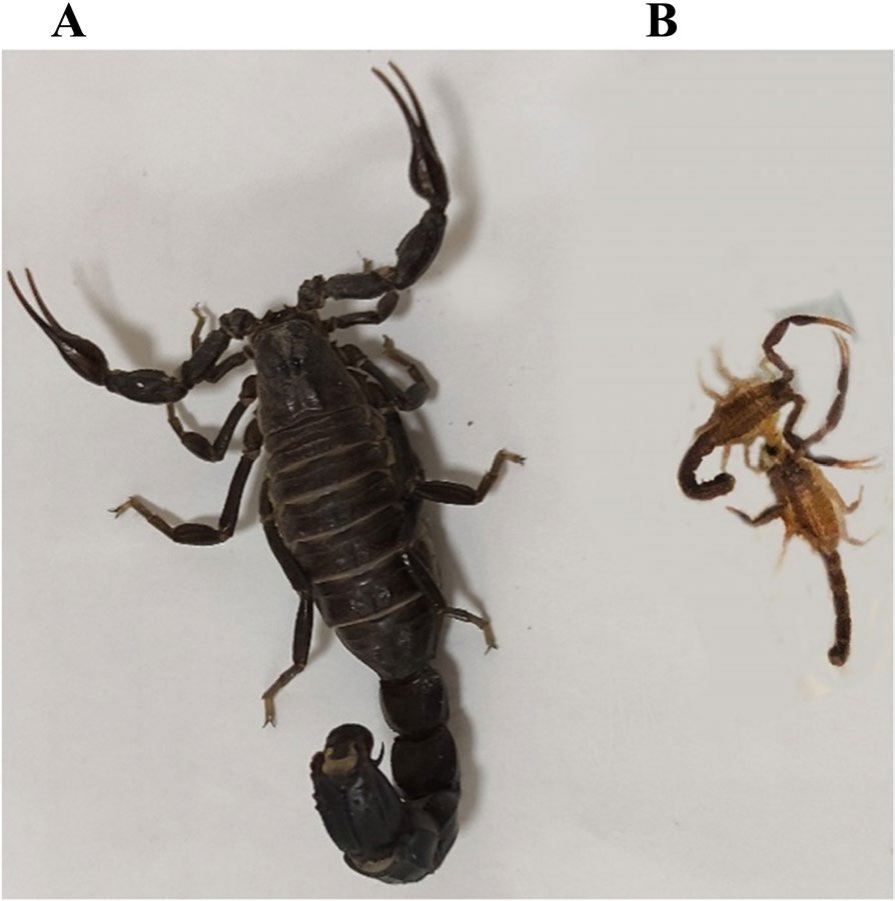
Dorsal view of *A. crassicauda* (**A**) adult (**B**) juvenile clearly illustrates differences of growth stages in this species. juveniles have smaller, brighter and more slender bodies in comparison to the adults, which have larger and black bodies

In Iran, based on cladistic morphological analysis, the genus *Androctonus* can be phylogenetically divided into 5 species; *A. crassicuda, A. baluchicus, A. amurexi, A. robustus*, and *A. finitimus* [11–13]. The scorpion *A. crassicauda* is one of the most venomous members of the family Buthidae. It is widely distributed in the Middle East countries, including Iran, Iraq, Turkey, Jordan, and Saudi Arabia [14]. To date, a lack of transcriptomic information is evident in the genus *Androctonus* and the available transcriptome data mostly concentrate on a few species in this genus, such as *A. bicolor* [15] and *A. mauretanicus* [16]*.* Furthermore, the comparative transcriptomic analysis of different growth stages of the scorpion has not been investigated so far. In the present study, we performed comparisons between venom glands from juvenile and adult stages of *A. crassicauda* scorpions (Fig. 1), with insights into the identification of the key genes associated with venom gland development. For this purpose, the gene expression profiles of the venom glands of adult and juvenile male scorpion *A. crassicauda* were comparatively analyzed. Finally, the potential functions of differentially dysregulated mRNAs were annotated by analysis of Gene Ontology (GO) and Kyoto Encyclopedia of Genes and Genomes (KEGG).

## Methods

### Sample collection and cDNA library construction

*A. crassicauda* scorpions were collected from the deserts of Baghmalek southwest of Khuzestan providence, Iran. After the classification of specimens according to morphological characteristics [17], the scorpions were grouped based on gender and age class as suggested by Nime et al. [18]. Thereafter, the telsons of 6 *A. crassicauda* individuals (3 adult males and 3 juvenile males) were removed 72 h after being milked by electrical stimulation. For total RNA extraction, the telsons were powdered in liquid nitrogen and total RNA was extracted with RNeasy Animal Mini Kit (Qiagen, Valencia, CA, USA) according to the manufacturer’s instructions. Then, the RNA Integrity Number (RIN) was evaluated for all samples with an Agilent Bioanalyzer 2100. Only RNA samples with a RIN > 7 were used for cDNA library construction. Finally, a total of 6 rRNA-depleted libraries were prepared for three independent biological replicates of adult males and juvenile males using the TruSeq Stranded Total RNA library preparation kit according to the manufacturer’s instructions (Macrogen, Seoul, Korea). Afterward, cDNA libraries were sequenced by the Illumina HiSeq 2000 platform (Illumina, San Diego, CA, USA), with 150 bp paired-end reads.

### Assembly of read-sequences and bioinformatics analysis

After cleaning, trimming, and assessing the quality of raw reads by means of the Trimmomatic and FastQC program (http://www.bioinformatics.bbsrc.ac.uk/projects/fastqc/), clean reads of 6 individuals were assembled de novo using Trinity software, version 2.0.3 [19, 20] on optimized parameters including –normalize_reads, –seqType fa, –SS_lib_type RF, –max_memory 32G, –CPU 8. The resulting transcriptome was generated in fasta format.

TransDecoder v3.0.1 with the ‘single best ORF’ option was used to predict the Open Reading Frames (ORFs) and potential coding sequences [20] within the generated transcriptome. Afterward, in order to identify transcripts harboring any protein domains in the de novo assembled transcripts/contigs, we used an annotation pipeline with five search strategies; Trinotate (http://trinotate.sourceforge.net), Blastp (http://blast.ncbi.nlm.nih.gov/Blast), Blastx (http://blast.ncbi.nlm.nih.gov/Blast), HMMER/Pfam (http://hmmer.janelia.org/software) and species-specific annotation filter. The main step consists of the identification of all putative homologous sequences with sequence similarity search against Swissprot, NCBI non-redundant (Nr), UniProtKB/TrEMBL, and Pfam protein domain databases with an E-value threshold of 10^−3^. Since the molecular identity of most scorpion venom short peptides (< 3001 Da) characterized using proteomic studies have not yet been discovered, it is not expected to determine many scorpion venom peptides by annotation against public databases. Therefore, we performed species-specific annotation to include novel scorpion toxin sequences and to identify many known homologs of proteins in animals with close phylogenetic affinity with scorpions.

We first created databases using the scorpion, tick, and spider specified protein sequences (https://www.uniprot.org/) and all manually reviewed venom proteins and toxins from the venomous animals (https://www.uniprot.org/program/Toxins). Then, the scorpion datasets were annotated manually with these sequences using blastx. Ultimately, all the annotated and identified coding transcripts were merged and known as mRNA transcripts.

### Quantification of gene expression levels of mRNAs

RSEM is an abundance estimation method for quantifying the gene and isoform expression levels in species with or without sequenced genomes that directly uses Bowtie to align the reads to the transcriptome [21]. RSEM software was used to estimate the gene expression levels in terms of FPKM (fragments per kilobase of transcript sequence per million base pairs sequenced). To quantify the gene expression levels in terms of FPKM in each individual, we aligned the clean paired-end reads for each of the six individuals (three adults and three juveniles) to the de novo assembled Trinity transcripts using the Bowtie alignment program [21, 22]. Then we used RSEM software to compute the number of reads mapped to each transcript on default values. RSEM uses the gap-free alignment produced by Bowtie as an input.

### Differential expression analysis of mRNA

Differential expression analysis of mRNA transcripts was determined using the edgeR package [22]. In multifactor RNA-Seq experiments, edgeR provides a generalized linear model (GLM) likelihood ratio test to calculate *p* values. The log ratio of fold change (logFC) ≥ 2 or logFC ≤  − 2 and *p-*value < 0.01 or FDR ≤ 0.05 were considered as the significance threshold to determine differentially expressed genes (DE) between the two groups.

To visualize the fold change and statistical significance of expression profiles for experimental groups, Volcano plot, and hierarchical clustering heatmaps analysis were represented by “ggplot2” and “Heatmap” R packages, respectively.

### Isolation and classification of DE venom gland proteins

The DE mRNA transcripts were isolated from all mRNA sequences and broadly grouped into different categories based on Pfam domains (https://pfam.xfam.org/).

### Gene ontology and pathway enrichment analysis

Differentially expressed coding genes in both growth stages were enriched for Gene Ontology (GO) and the Kyoto Encyclopedia of Genes and Genomes (KEGG) pathways (www.kegg.jp/kegg/kegg1.html) to investigate the main functions of DE mRNA. Further, GO and KEGG analyses also were conducted to interpret the possible functions by forming hierarchical categories based on the biological process, cellular component, and molecular function terms. The false discovery rate (FDR) <0.01 was considered significantly enriched.

## Result

### High-throughput sequencing and identification of candidate mRNAs

**Fig. 2:**
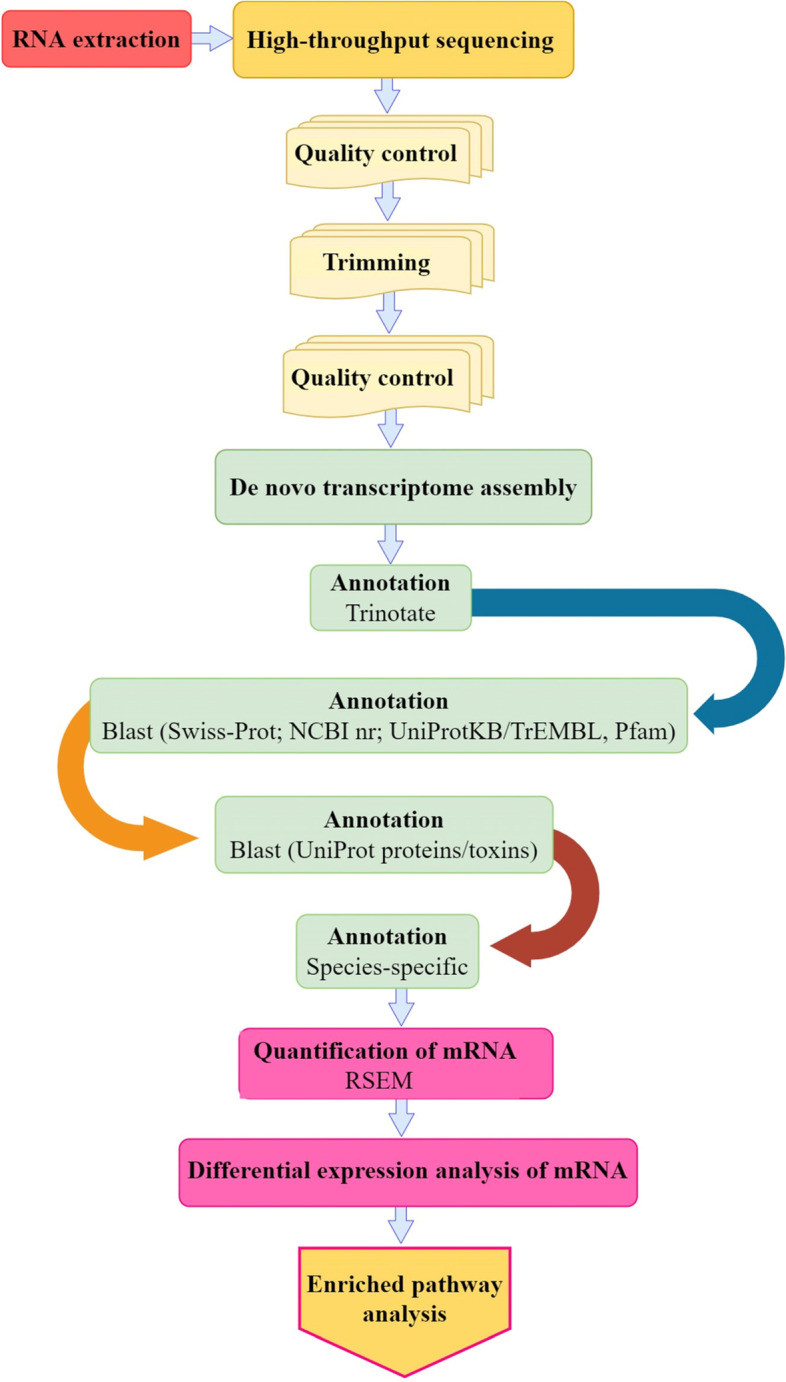
Overview of experimental and computational analyzing workflow. Briefly, the workflow involved stepwise filters based on annotations, gene expression, Differential expression analysis, and GO term and KEGG pathway enrichment analysis

**Fig. 3:**
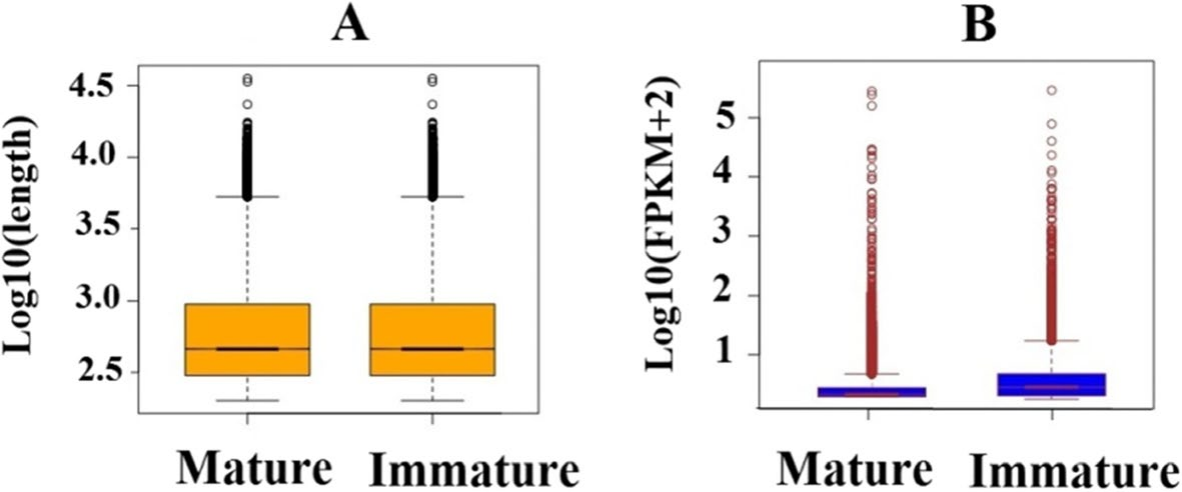
Characterization of *A. crassicauda* mRNA. **A** Length distribution of mRNA transcripts of adult and juvenile **B** Expression levels of mRNAs transcripts of adult and juvenile

To investigate changes in the venom gland gene expression among different developmental stages of *A. crassicauda*, an experimental workflow was implemented (Fig. 2). Briefly, paired-end sequencing of cDNA libraries was constructed. An average of 104,488,530 and 96,056,408 raw reads were gained for the venom glands of adult and juvenile scorpions, respectively. After filtering the raw reads and removing the low quality, adapter, and uncertain reads, a total of 104,484,814 and 96,052,932 clean reads, approximately 28 Gb and 26 Gb of high-quality data, were generated for the venom glands of adult and juvenile scorpions, respectively. The clean reads were assembled into 952,725 contigs after the removal of sequences shorter than 200 bp as uninformative using the Trinity tool on optimized parameters. TransDecoder v3.0.1 was used to extract all predicted protein-coding transcripts of venom gland transcriptome from *A. crassicauda*, and then to select the single-best open reading frame (ORF) (–single_best_orf) per assembled transcripts. After ORFs with less than 200 bp in length were removed from the assembly, the Trinotate pipeline was used to annotate the venom gland transcript ORF dataset. Then, similarity searches for all known organisms in Swiss-Prot; NCBI nr; UniProtKB/TrEMBL, Pfam, UniProt proteins/toxins databases, and species-specific annotation with blast were performed using nucleotide transcripts and protein sequences. After removing non-coding RNAs from the assembled transcriptomes, we annotated 148,992 coding sequences on the basis of blast hits to the Swiss-Prot; NCBI nr; UniProtKB/TrEMBL, and Pfam database. Of annotated sequences, a total of 38 coding sequences were predicted using TransDecoder, and 58,045 putative transcripts were found with sequence similarity to annotated genes from arachnids, 2,661 of which were scorpion-specific. Using the UniProt proteins/toxins database, we annotated an additional 215 venom proteins. All annotated sequences of each database were merged and considered as mRNAs. Finally, out of 952,725 transcripts, 209,951 were classified as mRNA transcripts. The comparison of the length of mRNA sequences of the two growth stage groups is illustrated in Fig. 3A. Sequence analysis suggested that mRNA candidates of both groups exhibited an equal sequence length (mean length of 838.77 bp). Furthermore, this study provided the first comprehensive analysis of the mRNA expression profiles in juvenile and adult scorpion venom glands (Fig. 3B). As shown in Fig. 3B, the gene expression levels differed significantly between candidate mRNAs of adult and juvenile groups. The expression of mRNAs of adult scorpions is lower compared with mRNAs of juvenile groups.

### Differential expression analysis of mRNAs

**Fig. 4:**
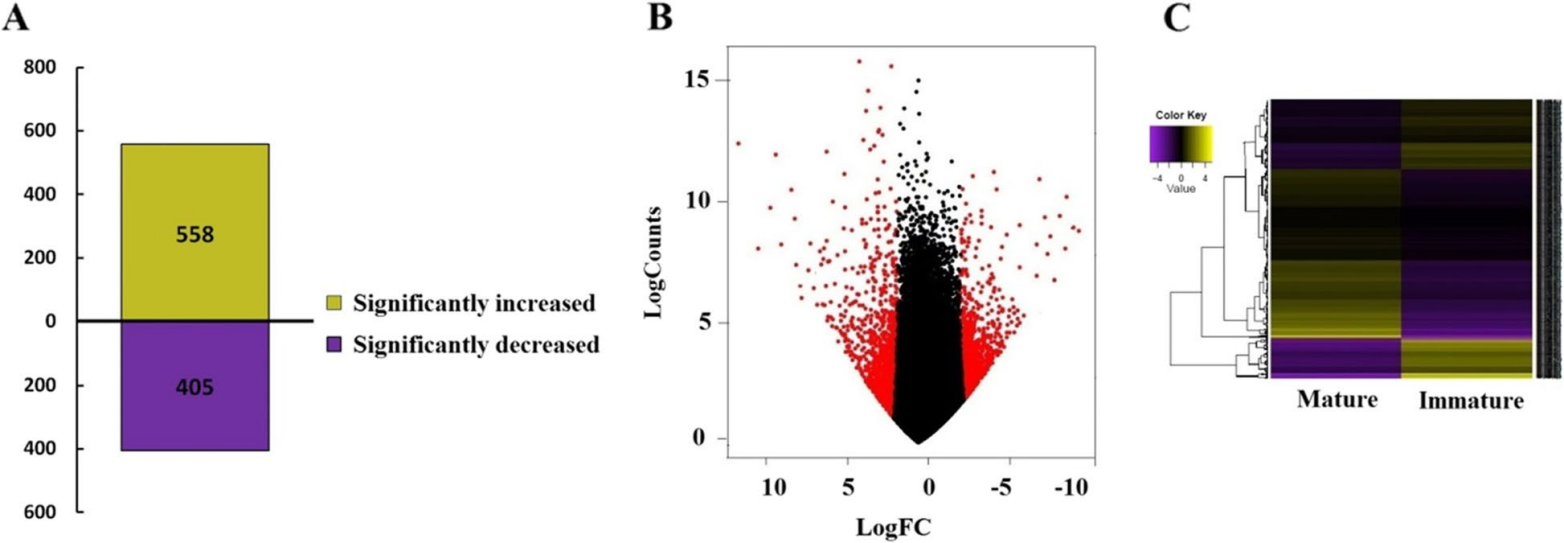
The distribution of fragments per kilobase of transcript per million (FPKM) values and visualization of differentially expressed mRNAs in adult and juvenile groups. **A** A Comparison of the up-regulated and down-regulated mRNAs **B** The Volcano plot of mRNA differential expression in adult and juvenile scorpions. Red and blank points refer to those differentially and not differentially expressed genes compared to adult scorpions (logFC ≥ 2 or logFC ≤  − 2 and *p*-value < 0.01). The scatter plot is a visualization method used for showing differentially expressed. **C** The heatmap of hierarchical clustering analysis. Heatmap showing the comparison of the gene abundance in the two groups of adult and juvenile. Colors from purple to yellow represent the gene expression abundance from downregulation to upregulation, respectively

To study the differential expression of venom gland mRNAs in the two developmental stages of *A. crassicauda*, the expression levels of mRNA transcripts were measured, and differential expression analysis was conducted using edgeR. 963 mRNA transcripts were identified with significant expression level differences (logFC ≥ 2 or logFC ≤ −2 and *p*-value <0.01 or FDR ≤0.05). Among these, 558 mRNAs were up-regulated, and 405 were down-regulated by adults (Fig. 4A). Volcano plot and heatmap of hierarchical clustering analysis were performed to visualize the fold change and determine the statistical significance of expression profiles for the adult scorpions after normalization to the juvenile scorpions. The Volcano plot and heatmap of hierarchical clustering analysis reveal differential gene expression patterns in the venom glands of adult and juvenile scorpions (Figs. 4B and C).

### Annotation of dysregulated mRNAs

**Fig. 5:**
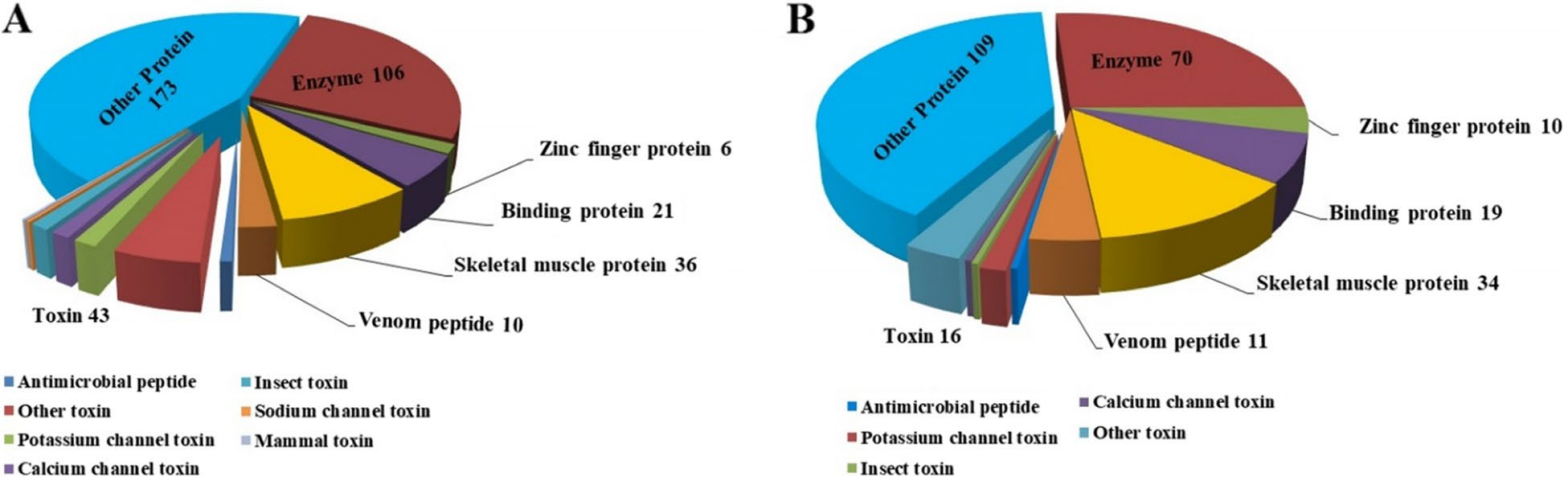
Classification of up and down-regulated mRNAs in adult scorpions compered to juvenile into venom components. **A** Relative distribution of the up-regulated genes identified in the venom gland transcriptome of adults *A. crassicauda*. The up-gulated toxic proteins were subdivided into distinct groups, including Ion channel toxins (12), Insect toxins (5), Mammal toxins (1), Antimicrobial peptides (3), and Other toxins (22). The number of transcripts classified for Potassium channel toxin, Calcium channel toxin and Sodium channel toxin are 6, 5 and 1, respectively. **B** Relative distribution of the down-regulated genes identified in the venom gland transcriptome of adult *A. crassicauda*. The down-gulated toxic proteins were subdivided into distinct groups, including Ion channel toxins (5), Insect toxins (1), Antimicrobial peptides (1), and Other toxins (9). The number of transcripts classified for Potassium channel toxin and Calcium channel toxin are 4 and 1, respectively

All differentially dysregulated mRNA sequences from each group were initially annotated on the basis of blast hits to the UniProt proteins/toxins and Swiss-Prot databases. To remove the redundancy occurring in the isoforms of the same genes, blast hits of 98% coverage and >99.0% identity were detected using the BLASTP program. After filtering the redundancy, we identified a total of 397 out of 558 up-regulated transcripts and 269 out of 405 down-regulated transcripts in the venom glands of adult scorpions compared to juveniles. Next, all identified proteins from adult and juvenile scorpion venom glands were classified based on the similarity of Pfam domains. The search for matching domains included not only scorpions but also all venomous animals and those showing a close phylogenetic affinity with scorpions. Seven identified categories include; Enzymes, Skeletal muscle proteins, Toxins, Binding proteins, Venom peptides, Zinc finger proteins, and Other proteins. The number of transcripts classified for each class is summarized in Fig. 5A and B. The major categories were further divided into subcategories. Below, we further describe those categories.

### Enzymes

Among up-regulated mRNAs, 106 recognized protein sequences have belonged to the enzyme classification. Of these, the most important enzymes are proteases (metalloproteinases (8) and serine proteases (5)), synthases, nucleotidases, oxidases, and aldehyde dehydrogenase (Fig. 5A). Here, we further found approximately 26% (70) of the differentially down-regulated transcripts codifying for enzymes. Of these, the most important enzymes were various venom proteinases (metalloproteinases (2) and serine proteases (12)), transferases (10), kinases (9), polymerase, fatty acid synthases, fatty acid elongase, and ligases (Fig. 5B).

#### Skeletal muscle proteins

Skeletal muscle proteins are another class of dysregulated proteins. 36 skeletal proteins were up-regulated in venom glands of the adult group, and the most important of these are Vinculin, Actin, Myosin, Tensin, Dystrophin, Kinesin, Chitin synthases, Chitinase cytoskeletal, Papilin, transmembrane proteins, and other cuticular proteins. Moreover, cytoskeletal proteins of adult-specific rigid cuticular protein, cuticlin-1, cuticle protein 14, and cuticle protein 16.8 were also up-regulated in the venom glands of adult scorpions.

In the juvenile scorpion's venom gland, 34 skeletal muscle proteins were up-regulated. Most of these include the structural proteins of the cytoskeleton that maintain the tissue integrity and regulation of microtubule dynamics. Of these, Plectin, Striatin, Ankyrin 2 and 3, Rootletin, Actin, Myosin, Titin, Talin-2, and Filamin-A cytoskeletal proteins can be mentioned.

#### Toxins

In this study, the dysregulated toxic proteins were subdivided into distinct groups, including Ion channel toxins, Insect toxins, Mammal toxins, Antimicrobial peptides, and Other toxins. As shown in Fig. 5, a total of 17 channel toxins were dysregulated, of which 12 channel toxins were up-regulated in the adult scorpion’s venom gland compared to juveniles, including one sodium channel toxin, five calcium channel toxins, and six potassium channel toxins. Whereas, a total of 5 channel toxins were down-regulated, including one calcium channel toxin and four potassium channel toxins.

Furthermore, a total of 3 antimicrobial peptides, 1 mammal toxin, 5 insect toxins, and 22 other toxin genes, such as toxin Acra (I-2, I-1, II-1, II-2 and III-1), U-scoloptoxins, Beta-toxin BmK, and other neurotoxins were significantly up-regulated by adult scorpions compared to juvenile, while a total of 1 insect toxin, 1 antimicrobial peptide, and 9 other toxin genes especially neurotoxins were significantly down-regulated (Fig. 5).

#### Other proteins

We identified a number of hemolymph proteins that become very highly expressed between adult and juvenile scorpion venom glands. Among interesting proteins that are up-regulated by adult scorpion venom glands, Hemocyanin, Hemocytin, Techylectin-5A, Hephaestin-like, Papilin, Sex determination protein tasselseed-2 and Dual oxidase maturation factor 1 can be mentioned (Fig. 5). Interesting proteins overexpressed by juvenile scorpions include Vitellogenin, Transforming growth factor-beta-induced protein ig-h3, and Endoplasmic reticulum lectin 1 (Fig. 5).

### GO terms and KEGG pathways enrichment analysis of DE mRNAs

**Fig. 6:**
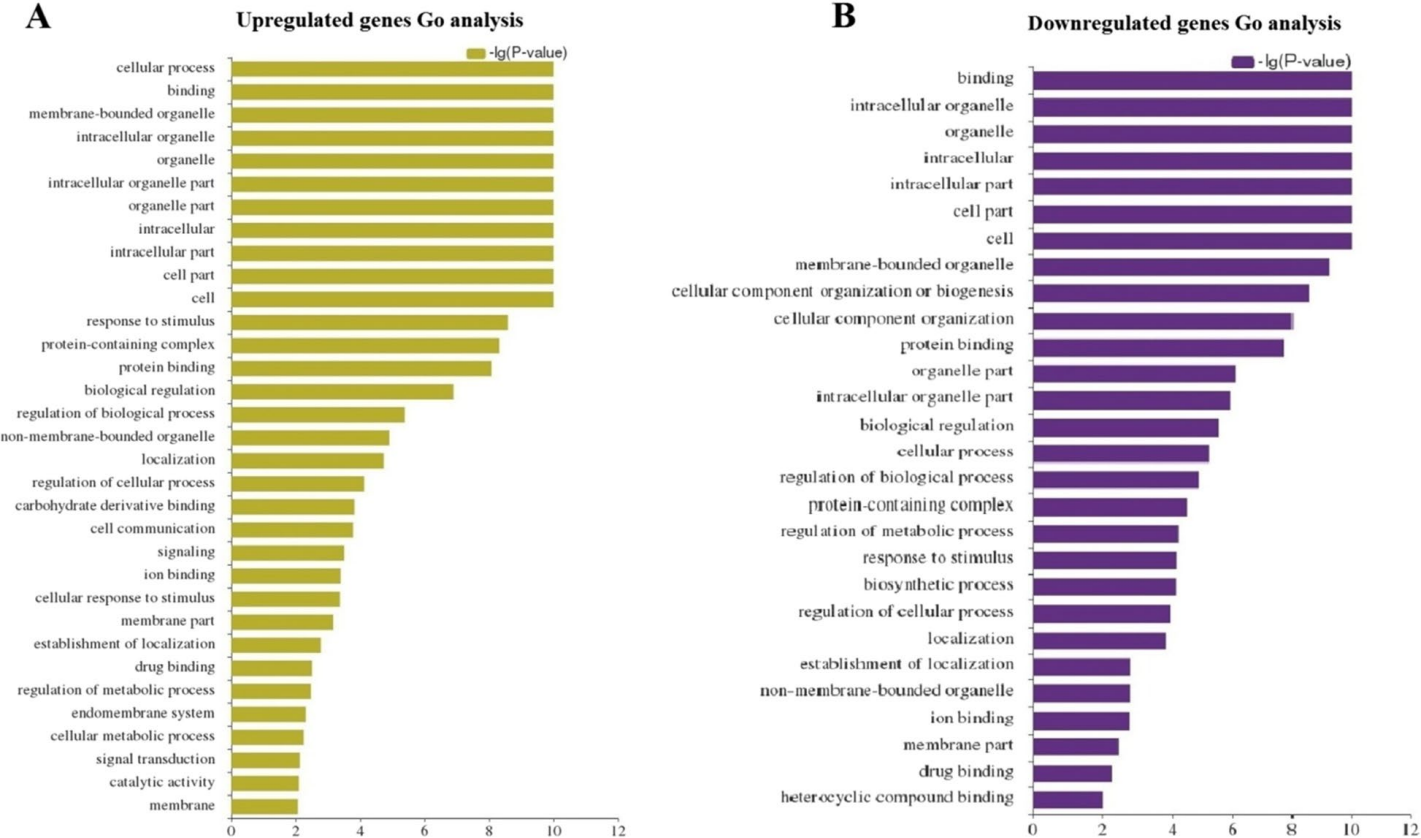
Gene Ontology (GO) analysis of the differentially expressed mRNAs from our dataset. **A** GO analysis of up-regulated mRNAs. **B** GO analysis of down-regulated mRNAs

The Gene Ontology (GO) and KEGG pathway enrichment analysis were carried out to explore the biological functions and pathways of DE mRNAs and to investigate gene groups that are participate in common biological responses or possess related functions. For the GO classification, the identified DE mRNAs were divided into three ontologies; molecular function, biological process, and cellular component. The GO analysis of differentially up-regulated mRNA transcripts (*p* < 0.01), revealed that “binding”, “catalytic activity” and “protein binding” in molecular function; “membrane”, “cell” and “cell part” in cellular component and “cellular process”, “organic substance metabolic process” and “cellular metabolic process”, in the biological process were highly represented (Fig. 6A, Supplementary file 1A). Correspondingly, our GO analysis result indicated that the most enriched GO terms of down-regulated genes in biological processes were mainly involved in “cellular process”, “biological regulation”, “regulation of biological processes”, “regulation of cellular processes”, and “response to stimulus”. In terms of molecular function, the down-regulated mRNAs were enriched in “binding” and “cellular process”, and in the cellular component, they were enriched in “cell” and “cell part” (Fig. 6B, Supplementary file 1B).

**Fig. 7:**
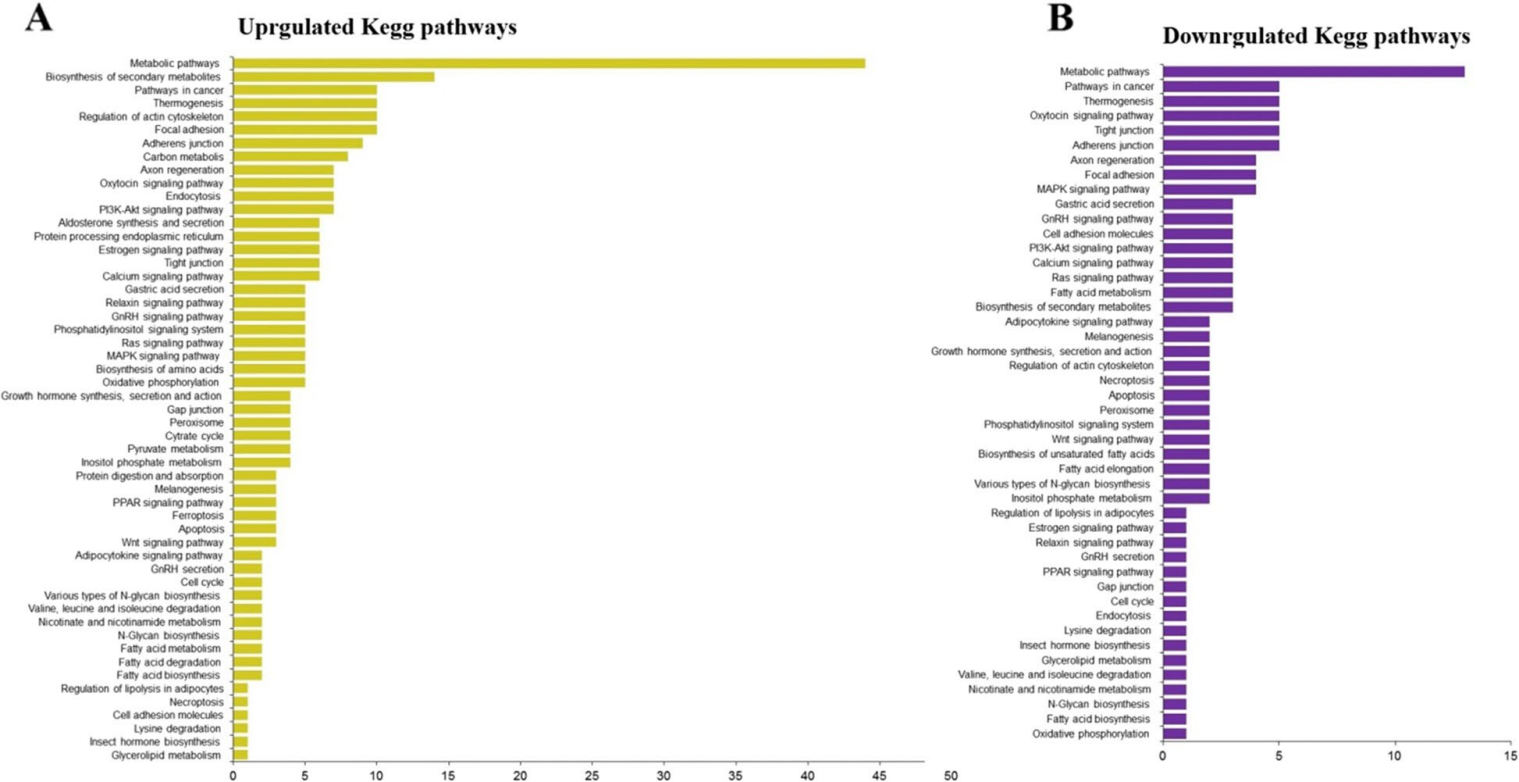
KEGG analyses of differentially expressed genes between the adult and juvenile groups. **A** The KEGG enrichment pathways for up-regulated mRNAs. **B** The KEGG enrichment pathways for down-regulated mRNAs. *P*-value cut-off was set at 0.01

KEGG analysis of the predicted DE mRNAs was used to enhance our knowledge of the biological functions of the DE mRNAs during the development of the *A. crassicauda* venom gland. KEGG analyses of differentially expressed genes between the adult and juvenile groups are shown in Fig. 7. According to KEGG analysis of differentially up-regulated mRNA transcripts, the most importantly enriched pathways were Metabolic pathways (44), Biosynthesis of secondary metabolites (14), Pathways in cancer (10), Regulation of actin cytoskeleton (10), Thermogenesis (10), Focal adhesion (10), Adherens junction (9), Carbon metabolism (8), Rap1 signaling pathway (8), Axon regeneration (7), Oxytocin signaling pathway (7), Endocytosis (7), PI3K-Akt signaling pathway (7), Estrogen signaling pathway (6), Calcium signaling pathway (6), GnRH signaling pathway (5), Gastric acid secretion (5), MAPK signaling pathway (5), Growth hormone synthesis, secretion and action (4), Melanogenesis (3), Fatty acid degradation (2), Regulation of lipolysis in adipocytes (1) and Insect hormone biosynthesis (1). In KEGG analyses of the down-regulated mRNAs, the most enriched pathways were Metabolic pathways (13), Pathways in cancer (5), Thermogenesis (5), Tight junction (5), Adherens junction (5), Axon regeneration (4), Focal adhesion (4), Oxytocin signaling pathway (4), MAPK signaling pathway (4), Gastric acid secretion (3), GnRH signaling pathway (3), PI3K-Akt signaling pathway (3), Calcium signaling pathway (3), Fatty acid metabolism (3), Biosynthesis of secondary metabolites (3), Melanogenesis (2), Growth hormone synthesis, secretion and action (2), Regulation of actin cytoskeleton (2), Apoptosis (2), Biosynthesis of unsaturated fatty acids (2), Fatty acid elongation (2) and Estrogen signaling pathway (1), respectively (Fig. 7). Regulatory pathways of the actin cytoskeleton, estrogen signaling, GnRH signaling, melanogenesis, and growth hormone synthesis, secretion, and action are illustrated in Supplementary file 2, Supplementary file 3, Supplementary file 4, Supplementary file 5, and Supplementary file 6 respectively.

According to the results of GO and KEGG analysis, chitin synthase (Chs), aminopeptidase and phosphodiesterase proteins were up-regulated proteins, and fatty acid elongase (Elovl) and fatty acid synthase proteins were the down-regulated proteins, which are the main enriched proteins in the metabolic pathway. Moreover, from up-regulated genes, the MYH, RDX, ACTN, VCL, PIP5K, and PP1C, and from down-regulated genes, the FGFR (Fibroblast growth factor receptor) were the main enriched proteins involved in the scorpion venom gland cytoskeleton development.

In the scorpion melanogenesis pathway, the up-regulated genes of GNAS, EGFR, CALM, and PLCB, and the down-regulated genes of PKA and CAMK were enriched. Furthermore, the up-regulated genes of GNAS, CREB, and PLCB, and the down-regulated genes of CACNA and PKA were enriched in the scorpion growth hormone signaling pathway. In the scorpion estrogen signaling pathway, the up-regulated genes of GNAS, EGFR, CREB, CoA, PLCB, and CALM, and the down-regulated gene of PKA were enriched. In the scorpion GnRH signaling pathway, the up-regulated genes of GNAS, EGFR, CREB, PLCB, and CALM, and the down-regulated gene of CACNA were the main enriched proteins. Taken together, the adults had severe gene upregulation compared to juvenile scorpions. In total, our results showed that the genes of Chs, MYH, RDX, ACTN, VCL, PIP5K, PP1C, GNAS, EGFR, CREB, CoA, PLCB, and CALM were up-regulated, and Elovl, CACNA, PKA, CAMK, and FGFR were down-regulated in adult scorpions.

## Discussion

So far, a comparative study of transcriptional profiles of adult and juvenile scorpion venom glands has not been performed. Here, a comparative transcriptome analysis of the venom gland of *A. crassicauda* scorpions in two different growth stages: juvenile and adult, was done to identify the key genes for scorpion venom gland development. For this purpose, the scorpion venom gland gene expression level was estimated using the FPKM value [21] and the differentially expressed genes during venom gland development of *A. crassicauda*, were identified based on the experimental workflow shown in Fig. 2. Using this workflow, we found 963 (558 up-regulated and 405 down-regulated mRNAs in venom glands of adults compared to the juvenile scorpions) out of 209,951 mRNA transcripts that were differentially expressed. In recent years, a number of high-throughput venom gland transcriptome studies on scorpions have divided the venom components into functional classes [23–25]. Here, to determine the function of dysregulated genes, the up and down-regulated mRNAs were also classified into several subclasses. We have further described this classification below.

### Enzymes

We detected 106 out of 558 up-regulated mRNAs that showed enzyme activity, including proteases, synthases, nucleotidases, oxidases, and aldehyde dehydrogenase. Scorpion venom is well known as a rich source of different proteases [26]. Among the up-regulated proteases are metalloproteinases and serine proteases, which are important venom proteins for protein digestion, catalytic, post-translational processing of toxin activity, and promote the spreading of toxins via degradation of matrix protein activity that has already been described in scorpion venom [26, 27], snake venom [28] and spider venom [29]. Metalloproteinase is one of the common proteolytic enzymes of scorpion venom. Metalloproteinase enzymes have been reported to play critical roles in envenomation-related pathogenesis by affecting the hemostatic system, including bleeding, intravascular clotting, edema, inflammation, myonecrosis, skin damage and inflammatory reaction as an important characteristic of snake bites envenomation [30, 31]. Additional activities of these enzymes are in cell proliferation, differentiation, and remodeling of the extracellular matrix [25]. De Oliveira et al. [25] have suggested that metalloproteinase plays an important role in the pathogenesis of the local tissue damage during snake envenomation and may be associated with hemorrhage by disrupting the microvessel walls. Such observations may suggest that one of the causes of envenomation-related pathogenesis damage may be related to overexpression of these enzymes. It is noteworthy that these enzymes are highly overexpressed in adult scorpions, which may indicate higher toxicity of these scorpions. On the other hand, Aldehyde dehydrogenase (ALDH) is one of the up-regulated enzymes involved in important pathways such as Pyruvate metabolism, Fatty acid degradation, and Insect hormone biosynthesis. Aldehyde dehydrogenase is a biosynthetic enzyme of the Juvenile hormones (JH) pathway that plays a critical role in insect morphogenesis, reproduction, and behavior [32]. In general, the number of enzymes overexpressed in adult scorpions (106) is higher than in juvenile ones (69). This may suggest higher enzymatic activity in adult scorpion venom glands.

Down-regulated proteins were also classified according to up-regulated proteins classification. Of differentially down-regulated mRNA, 70 out of 269 annotated transcripts were found to be enzymes. Interestingly, the obtained results of differentially expressed enzymes showed that the major up-regulated synthase enzymes of adult scorpions catalyzed the synthesis of amino acid, phosphate, and citrate compounds, while the major synthase enzymes which overexpressed in juvenile scorpions include: fatty acid synthases and fatty acid elongase. It has been reviewed that the expression of fatty acid elongation proteins led to increases in very long chain fatty acid synthesis, which is widespread among insect taxa. Very long chain fatty acids are linked with other compounds to produce biologically active products that are involved in the biosynthesis of cuticular waxes, and they have an important role as anti-inflammatory components [33–36]. Fatty acid elongation protein and elongation of very long chain fatty acid protein is essential in the biosynthesis of fatty acids longer than C14. These enzymes are involved in the pathway of fatty acid biosynthesis, which is part of lipid metabolism and may be required for normally rapid growth [37]. Our results are consistent with findings in mealworms that determined the expression of two different elongases throughout all developmental stages [37]. Of other down-regulated enzymes, Transferase enzymes can be mentioned that are generally known to catalyze the transfer of L-fucose sugar, glutaminyl-peptide, acetyl, methyl, and galactosamine from a donor substrate to an acceptor compound.

### Skeletal muscle proteins

Furthermore, 36 out of 558 up-regulated mRNAs that encode many skeletal muscle proteins were recognized. Of these proteins, the most important are Vinculin, Actin, Myosin, Tensin, Dystrophin, Kinesin, Chitin synthases, Chitinase cytoskeletal, Papilin, transmembrane proteins, adult-specific rigid cuticular protein, cuticlin-1, cuticle protein 14 and cuticle protein 16.8. Insect cuticles form an exoskeleton that has a rigid structure due to the presence of chitin and sclerotized proteins. Chitin is a vital polysaccharide of the cuticular exoskeletons and the peritrophic matrix of arthropods, which is synthesized by a complex family of Chitin synthases and degraded via Chitinases. Chitin is considered primarily a structural scaffold protein supporting the cuticles of the epidermis and trachea as well as the gut epithelium peritrophic matrices. Insect development and morphology forces them to periodically replace their old cuticles with new ones during molting. Therefore, in order to coordinate synthesis and degradation of the cuticle, insects consistently require strict control of the participating components during molting, such as Chitin synthases, Chitinase, Actin, Myosin and Kinesin. To achieve this goal, insects regularly produce Chitin synthases, Chitinase enzymes, Actin, Myosin and Kinesin during development [38–40]. Furthermore, Vinculin and Kinesin have been reported to up-regulate upon differentiation and they may be involved in the differentiation of adult muscle cells [41–43]. Chitinase is also investigated to have neurotoxic effects include an important role in the pathogenesis of inflammatory and allergic disease conditions [44, 45].

Dystrophin, a member of the β-spectrin/α-actinin protein family, is a cytoskeletal protein that is located in the muscle sarcolemma [46]. Dystrophin is part of a protein complex that works together to maintain the muscle fibers by connecting the cytoskeleton to the basal lamina and protecting them from injury [47]. Based on this study results, adult skeletal muscle consists of highly cytoskeletal proteins that strengthen the muscle fibers and regulate muscle cell activity. Accordingly, Ginkel and Wordeman [43] reported the presence of Kinesin in differentiating and adult skeletal muscles.

In juvenile scorpions, 34 skeletal muscle proteins out of 269 annotated transcripts were up-regulated. The most important ones are Plectin, Striatin, Ankyrin 2 and 3, Rootletin, Actin, Myosin, Titin, Talin-2 and Filamin-A. To date, scorpion skeletal muscle protein’s function has not been explored. In eukaryotic cells, Ankyrins are intracellular adaptor proteins critical for the expression and targeting of cytoskeletal elements that serve as the interface between the plasma membrane and cytoskeleton [48]. Previous studies have suggested an important role for Ankyrin protein during embryonic development [49]. Similar to our results, Daniel et al. [49] showed that both forms of Ankyrin were ubiquitously expressed early in development stages.

Plectin, a large size (>500 kDa) cytolinker protein, is abundantly expressed in a wide range of cells and tissues, most prominently in muscle, brain and stratified squamous epithelia [50]. It acts as a multi-functional cytoskeletal intermediate filament (IF)-associated protein and signaling scaffold [51]. There is little information about the expression of this protein in different stages of life, especially in insects, and comparing its expression levels in different life stages requires further study.

Striatin, a multivalent scaffold protein, was found to act as a regulator of calcium homeostasis [52]. In rats, the highest expression of Striatin occurs at neonatal stages and the lowest in adults [53] as we also observed for *A. crassicauda*.

Rootletin has been shown to be one of the linker proteins that is the only structural constituent of the ciliary rootlet [54]. The Ciliary rootlet is a prominent fibrous cytoskeletal structure that originates from basal bodies of ciliated cells and links the base of the cilium to the cell body [55, 56]. Previous studies have reported the presence of sensory organs (sensory hairs) in various parts of the scorpion cuticle, including the pedipalps, legs, pectin and venom gland [57–59], but no study has been done on their structural proteins in scorpions so far. The function of Rootletin has already been investigated in the fruit fly. The previous studies have shown that Rootletin is essential for mechanosensory function and ciliary rootlet formation in chordotonal sensory neurons, and it could organize the ciliary rootlet to achieve neuron sensory function that affecting various behaviors related to mechanosensation and chemosensation in *Drosophila* [55, 60]. Considering the various vital physiological roles of cilia such as; chemoreception, mechanoreception, hygroreception and thermoreception in arthropods [61]; motility, sensory and signalling roles in insects [62]; chemosensory and mechanosensory in scorpions [57, 63, 64] the study of the expression of this protein seems to be necessary. Our knowledge about the function and expression pattern of this protein as well as the roles of sensory cilia in the scorpions is limited and needs more attention. Although interestingly, the results of this study showed that this protein is expressed in two investigated stages of the scorpion life cycle and the obtained results indicated an increase in its expression in juvenile scorpions compared to adult ones. However, the expression pattern of Rootletin in scorpions requires further studies.

Titin is a large scaffold protein in striated muscle physiology [65]. In previous literature, Titin is also called mini-titin, titin/twitchin, titinlike, connectin, etc [66]. Due to its springlike nature, Titin is suggested to fulfill multiple functions. It causes passive elasticity of muscle, maintains the thick filaments in the center of the sarcomere, provides much of muscle passive resistance to stretch and elasticity and determines myofilament distensibility. In addition, it regulates the activity of other sarcomere proteins and preserves myosin molecules in place. Due to its elasticity, this protein may help determine the length of the sarcomere during development [66, 67], which may be the reason for the upregulation of these proteins in juvenile scorpion’s venom glands.

Talins are large, cytoskeletal proteins found in animals that link the actin cytoskeleton to the plasma membrane in several multicomponent adhesion complexes [68, 69]. Talin-2 is crucial for myoblast fusion, multicellular differentiation and morphogenesis during skeletal muscle development [68, 70, 71]. We found that Talin-2 is highly expressed in juvenile scorpions.

Dystroglycan is a laminin binding glycoprotein that serves as a transmembrane linker between intracellular cytoskeleton and extracellular matrix protein [72, 73]. Recently, it was suggested that Dystroglycan is expressed in developing epithelial cells and may be important for the maintenance of tissue integrity [72, 74]. Dystroglycan is also required for the formation of a basement membrane at the early stages of development [75]. In this study, we found that Dystroglycan is over expressed in juvenile scorpions.

### Toxins

Venom from scorpions consists of a rich source of polypeptide toxins, which are classified into Ion channel toxins, Insect toxins, Mammal toxins, Antimicrobial peptides, and Other toxins based on primary targets and their toxicity to insects, or mammals, or other kinds of organisms. Of these polypeptide toxins, ion channel toxins are known to be neurotoxins, which bind to different specific receptor sites of voltage dependent ion channels with high affinity and modulating voltage-gated ion channel activity. These compounds lead to high toxicity and neurotoxic symptoms of scorpion venom during envenomation [25, 76].

Here, we compared the overexpression of toxic proteins of adult and juvenile scorpion venom glands. We found that the number of up-regulated toxins in adult groups is more abundant than juvenile scorpions. As shown in Fig. 5, Supplementary file 7 and Supplementary file 8, several insects, mammals, antimicrobial specific venom toxins, ion channel toxins and other toxins have been overexpressed in adult scorpion venom glands. However, no mammal specific venom toxin was found in juvenile scorpions. Differences in the number of identified specific venom toxins may suggest that the venom of adult scorpions is more toxic to insects, mammals and other organisms than juvenile ones.

Moreover, 12 and 5 channel toxins were up and down-regulated by adults compared to juvenile scorpion’s venom glands, respectively. Interestingly, as shown in Fig. 5, the majority of the differentially expressed channel toxins are codifying for potassium channel toxins. Potassium channels, a family of neurotoxins, ubiquitously exist in excitable and nonexcitable cells. Potassium channels can be divided into four major classes based on their functionality and structure: calcium activated, voltage-gated, inward rectifier, and two-pore K^+^ channels [77]. Most of the putative potassium channel toxins described here have similarities to the voltage-gated potassium channel-blocking group and may be involved in scorpion envenomation. Adult scorpions express higher levels of potassium channel toxins. Therefore, causes of more symptoms following envenomation by adult scorpion venom may be due to increased expression of potassium channel toxins by the adult group compared to juveniles.

### Other proteins

Of up-regulated mRNAs by adult scorpions, we have identified hemolymph proteins including; Hemocyanin, Hemocytin, Techylectin-5A and Hephaestin-like proteins. Techylectin-5A is one of the most overexpressed proteins in adult scorpions. Recently, studies suggested that Tachylectins play an important role as pattern recognition molecules in the innate immune response system and blood coagulation [78]. Homocyanin, a large copper-binding metalloprotein in the hemolymph of invertebrates, acts as a respiratory pigment in the transport of oxygen and is responsible for the blue coloration of arthropods hemolymph [79, 80]. The present studies showed that, even though the levels of Hemocyanin mRNA were found in all samples, the highest level of Hemocyanin mRNA expression occurred in the adult scorpions. Contrary to the results obtained in this study, it has been reported that a moderate amount of Hemocyanin was found in all developmental stages of the grasshopper species *Locusta migratoria manilensis* life cycle but the levels of Hemocyanin mRNA in the embryo were significantly higher than in adults [81].

The preliminary studies on the physiological properties and structure of Haemocyanin in scorpions have confirmed the respiratory and also buffering function of Haemocyanin [80, 82]. None of them have been compared the expression of Homocyanin in adult and juvenile scorpions and this issue needs further investigation.

Papilin, an extracellular matrix glycoprotein, is the other up-regulated protein of venom glands of adult scorpions. It has been suggested to influence cell rearrangements and may modulate metalloproteinases during organogenesis and development [83]. With this explanation, simultaneous overexpression of Papilin with Homocyanin proteins may confirm overexpression of each other.

Other up-regulated mRNAs in juvenile scorpions are Vitellogenin, Transforming growth factor-beta-induced protein ig-h3 and Endoplasmic reticulum lectin 1. Vitellogenin (vtg) is a phospholipoglycoprotein involved in egg yolk formation and produced by most oviparous organisms. In the majority of insects, Vtg is synthesized extraovarially in the fat body [84–87].

Previously, it was hypothesized that Vitellogenin is only expressed in females of social insects, but evidence has recently been obtained that Vtg is also expressed in males as well as in subsocial insects such as beetles [85]. In addition to its multiple social roles such as regulation of social behavior, Vtg has been shown to be involved in nutritional functions, antioxidant activities, immune defense, oocyte development, reproduction and longevity regulation [88–90]. Furthermore, Vtg production increases in male fish when exposed to exogenous estrogen, in mosquitoes when blood socking [87], in frogs, yellowfin seabream and roosters under estrogen stimulation [89, 91, 92], in hymenoptera males and females during parental care [85] and in honeybees during foraging behavior and primes bees for specialized foraging tasks [90].

Evidence shows that estrogen in males plays a critical role in sexual function, and it is essential for spermatogenesis. Moreover, the dynamic process of spermatogenesis in juvenile males is regulated by estrogen [89]. Most importantly, the presence of Vtg in scorpions has been reported in this investigation. Thus, this study reports both the expression of this protein and evaluated the difference in its expression between adult and juvenile scorpions. The biological significance of DE analysis has been shown that the expression of this protein is significantly increased in juvenile scorpions. Increases in Vtg expression in juvenile males, may be due to exogenous estrogen stimulation [89]. In this study, we evaluated some of the most important differentially expressed genes between adult and juvenile scorpions; refer to Supplementary file 3 and Supplementary file 4.

#### Gene Ontology of DE mRNAs

To identify gene groups that participate in the development of scorpion venom glands, a bridge was required to associate DE mRNAs and critical biological processes and molecular pathways. Dysregulated mRNAs from the venom glands of adult scorpions compared to the venom glands of juvenile scorpions are suitable for investigating gene groups that are participate in common biological responses or possess related functions. For this purpose, we analyzed each dysregulated mRNA for its contribution in GO terms and KEGG pathways and key GO terms and KEGG pathways were identified. Analysis of GO and KEGG were used to enhance our knowledge of the biological functions of the DE mRNAs [93] during development of the venom gland of *A. crassicauda*. Interestingly, this study GO and KEGG analysis revealed the top enriched GO terms and KEGG pathways of each DE mRNAs, which were highly enriched in the development of *A. crassicauda* venom gland, such as metabolic, thermogenesis, cytoskeleton, estrogen signaling, GnRH signaling, growth hormone signaling and melanogenesis pathways. Therefore, in this study, altering the expression pattern of genes involved in these pathways is suggested as key indicators of scorpion venom gland development.

Of these, some important genes, including the Chs, Elovl, MYH, RDX, ACTN, VCL, PIP5K, PP1C, FGFR, GNAS, EGFR, CREB, CoA, PLCB, CALM, CACNA, PKA and CAMK genes were suggested key regulation factors of development in scorpion venom gland. These GO terms and KEGG pathways can provide new insight for the study of scorpion venom gland development. Several other studies were also undertaken to elucidate the role of this important mediator in the reproductive processes of males. Leclerc et al. [94] have demonstrated that Calmodulin (CALM) is a small conserved protein that is known to be expressed abundantly in the testis, especially in the spermatozoa. This acidic protein mediates numerous intracellular Ca^2+^ dependent events, including sperm motility and fertilizing ability. In the present study, upregulation of calmodulin in adult scorpions further emphasizes the importance of this Ca^2+^ mediator in sperm function.

On the other hand, protein kinase A (PKA) and calcium/calmodulin-dependent protein kinase (CaMK) also have been investigated to regulate cellular growth, differentiation, sperm maturation and function. Furthermore, they are known to play a major role in spermatogenesis. Both protein kinase A (PKA) and CaMKIV can phosphorylate CREMτ (cAMP-responsive element modulator-τ), which is an essential transcription factor for spermatogenesis [95, 96]. The results of the present study can be further validated by a report by Ma et al. [97], who demonstrated that PP1C (serine/threonine-protein phosphatase pp1 catalytic subunit) involved in spermatogenesis and by Hasegawa et al. [98] who indicated that PIP5K1A (phosphatidylinositol-4 phosphate 5-Kinase) may play a role in the maintenance of sperm number and morphology during spermatogenesis.

## Conclusion

Recently, transcriptomic analyses of scorpion venom glands were carried out by high-throughput sequencing and further analysis, but so far, the transcriptional analysis of venom glands of scorpions in different growth stages (adult and juvenile) has not been examined. The lack of information about juvenile scorpion venom, as well as the differences between their venom components with adult scorpions, highlights the need for such studies. In the present study, a rich source of venom proteins was identified using the RNA-seq technique and using various databases for the isolation and identification of protein coding RNAs. Comparison of venom components of juvenile and adult scorpions using gene expression analyses identified DE genes and pathways significantly associated with scorpion growth. By analyzing DE genes, we suggested the Chs, Elovl, MYH, RDX, ACTN, VCL, PIP5K, PP1C, FGFR, GNAS, EGFR, CREB, CoA, PLCB, CALM, CACNA, PKA and CAMK genes as key regulators of scorpion growth. Taking our results together, these findings broadened our knowledge about the differences between adult and juvenile scorpion’s venom. Furthermore, this study provides new insights into the application of comparative transcriptome analysis to identify the special key genes for venom gland development.

## Supplementary Information

**Additional file 1: Supplementary file 1.** Gene Ontology analysis of differentially expressed mRNAs from scorpion dataset. (A) GO analysis of up-regulated mRNAs. (B) GO analysis of down-regulated mRNAs. **Supplementary file 2.** Regulation of actin cytoskeleton pathway. The up-regulated genes MYH, RDX, ACTN, VCL, PIP5K, PP1C, RAC1 and the down-regulated gene FGFR (Fibroblast growth factor receptor) were enriched in scorpion venom gland cytoskeleton development. Green color indicates up-regulated genes of adults. **Supplementary file 3.** Estrogen signaling pathway. The up-regulated genes GNAS, EGFR, CREB, CoA, PLCB and CALM the down-regulated gene PKA were enriched in scorpion estrogen signaling pathway. Green color indicates up-regulated genes of adults. **Supplementary file 4.** GnRH signaling pathway. The up-regulated genes of Gs (GNAS), EGFR, CREB, PLCß and CaM (CALM), and the down-regulated gene of CACNA were enriched in scorpion GnRH signaling pathway. Green color indicates up-regulated genes of adults. **Supplementary file 5.** Melanogenesis pathway. The up-regulated genes of GNAS, EGFR, CALM and PLCB, and the down-regulated genes of PKA and CAMK were enriched in Melanogenesis pathway. **Supplementary file 6.** Growth hormone synthesis, secretion and action pathway. The up-regulated genes of GNAS, CREB and PLCB, and the down-regulated genes of CACNA and PKA were enriched in scorpion growth hormone signaling pathway. **Supplementary file 7.** Classification of up-regulated mRNAs into venom components. **Supplementary file 8.** Classification of down-regulated mRNAs into venom components.

## Data Availability

The mRNA and protein sequences reported in this paper are appearing in the NCBI under the accession numbers OM296278, OM296279, OM296280, OM296281, OM296282, OM296283, OM296284, OM296285, OM296286, OM296287, OM296288, OM296289, OM296290, OM296291, OM296292, OM296293.

## Abbreviations

CACNA: Voltage-dependent calcium channel L type alpha
CALM: Calmodulin
CAMK: Calcium/calmodulin-dependent protein kinase (CaM kinase) II
Chs: Chitin synthase
CoA: Nuclear receptor coactivator 1
CREB: Cyclic AMP-dependent transcription factor ATF-4
DE: Differentially expressed
EGFR: Epidermal growth factor receptor
Elovl: Fatty acid elongase
FDR: False discovery rate
FGFR: Fibroblast growth factor receptor
GNAS: Guanine nucleotide-binding protein
GO: Gene ontology
KEGG: Kyoto Encyclopedia of Genes and Genomes
PIP5K: Phosphatidylinositol-4 phosphate 5-Kinase
PKA: Protein kinase A
PLCB: Phosphatidylinositol phospholipase C, beta
PP1C: Serine/threonine-protein phosphatase pp1 catalytic subunit
RAC1: Ras-related c3 botulinum toxin substrate
Vtg: Vitellogenin
