# Supplementary material for: Differential venom gland gene expression analysis of juvenile and adult scorpions Androctonus crassicauda
Source: BMC Genomics. 2022 Sep 8;23:636. doi: 10.1186/s12864-022-08866-1 (PMC9454214; doi:10.1186/s12864-022-08866-1)
Supplement: Supplementary file 1 — Additional file 1: Supplementary file 1. Gene Ontology analysis of differentially expressed mRNAs from scorpion dataset. (A) GO analysis of up-regulated mRNAs. (B) GO analysis of down-regulated mRNAs. Supplementary file 2. Regulation of actin cytoskeleton pathway. The up-regulated genes MYH, RDX, ACTN, VCL, PIP5K, PP1C, RAC1 and the down-regulated gene FGFR (Fibroblast growth factor receptor) were enriched in scorpion venom gland cytoskeleton development. Green color indicates up-regulated genes of adults. Supplementary file 3. Estrogen signaling pathway. The up-regulated genes GNAS, EGFR, CREB, CoA, PLCB and CALM the down-regulated gene PKA were enriched in scorpion estrogen signaling pathway. Green color indicates up-regulated genes of adults. Supplementary file 4. GnRH signaling pathway. The up-regulated genes of Gs (GNAS), EGFR, CREB, PLCß and CaM (CALM), and the down-regulated gene of CACNA were enriched in scorpion GnRH signaling pathway. Green color indicates up-regulated genes of adults. Supplementary file 5. Melanogenesis pathway. The up-regulated genes of GNAS, EGFR, CALM and PLCB, and the down-regulated genes of PKA and CAMK were enriched in Melanogenesis pathway. Supplementary file 6. Growth hormone synthesis, secretion and action pathway. The up-regulated genes of GNAS, CREB and PLCB, and the down-regulated genes of CACNA and PKA were enriched in scorpion growth hormone signaling pathway. Supplementary file 7. Classification of up-regulated mRNAs into venom components. Supplementary file 8. Classification of down-regulated mRNAs into venom components. [file 12864_2022_8866_MOESM1_ESM.docx]

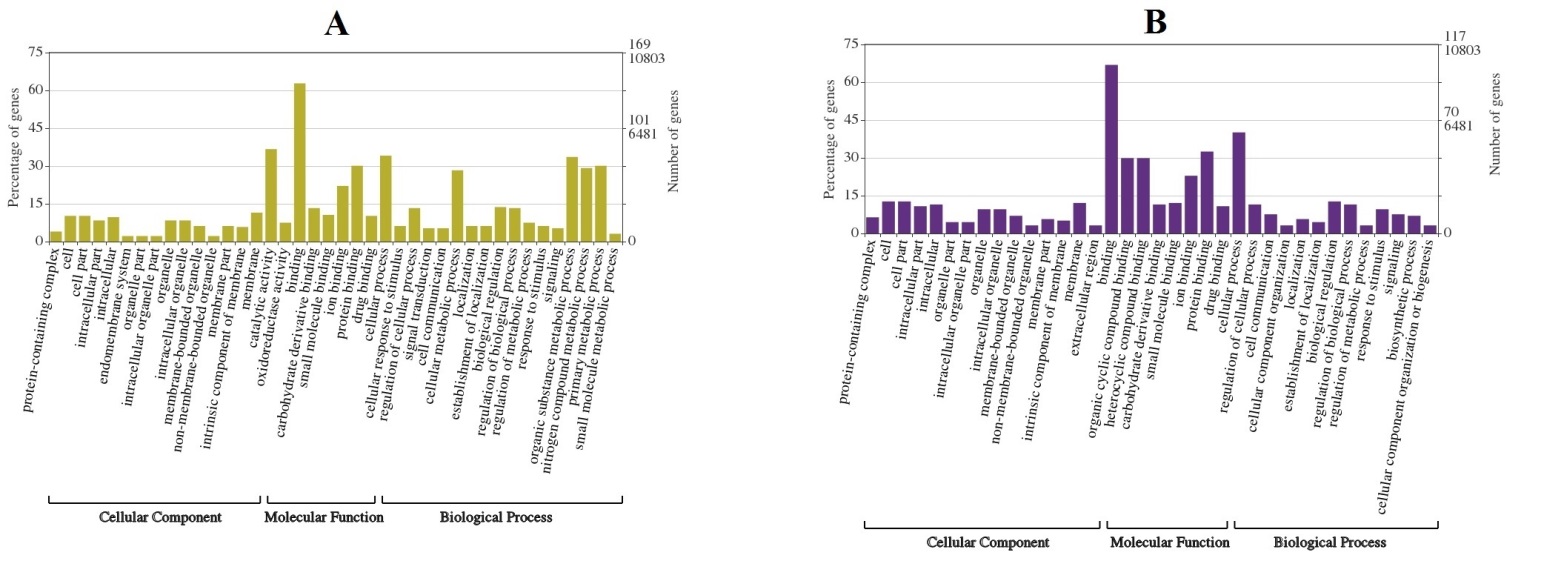


**Supplementary file 1.** Gene Ontology analysis of differentially expressed mRNAs from scorpion dataset. **(A)** GO analysis of up-regulated mRNAs. **(B)** GO analysis of down-regulated mRNAs.


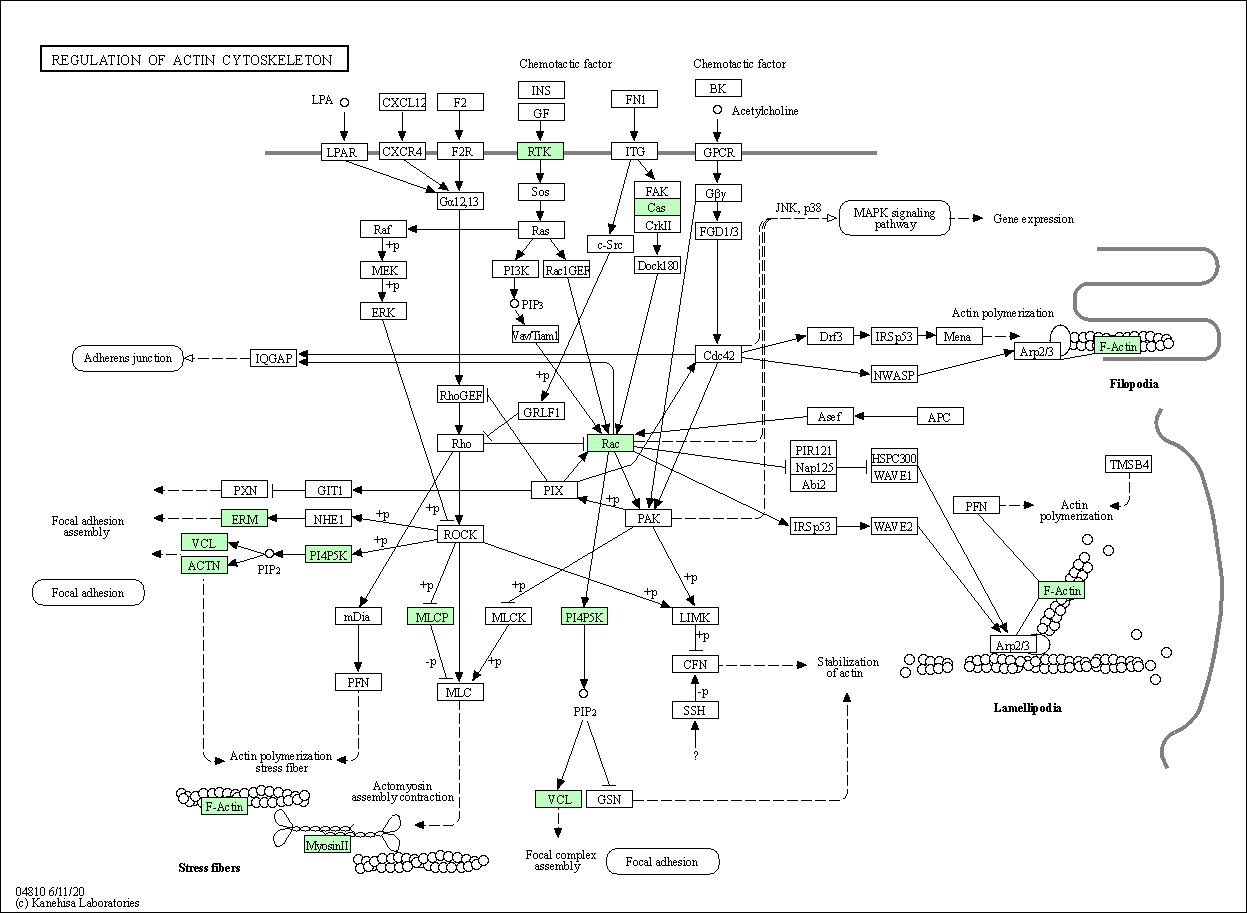


**Supplementary file 2.** Regulation of actin cytoskeleton pathway. The up-regulated genes MYH, RDX, ACTN, VCL, PIP5K, PP1C, RAC1 and the down-regulated gene FGFR (Fibroblast growth factor receptor) were enriched in scorpion venom gland cytoskeleton development. Green color indicates up-regulated genes of adults.


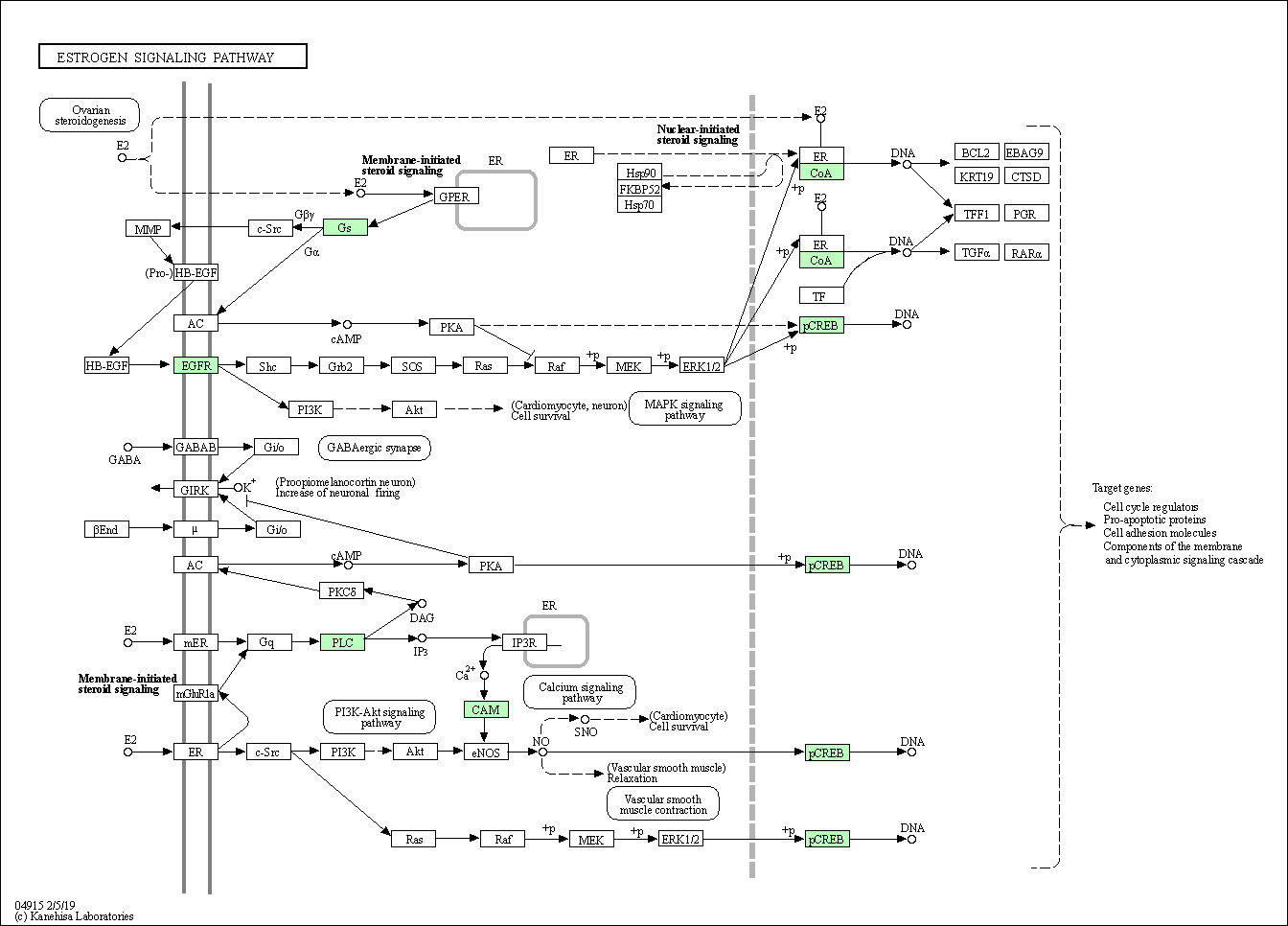


**Supplementary file 3.** Estrogen signaling pathway. The up-regulated genes GNAS, EGFR, CREB, CoA, PLCB and CALM the down-regulated gene PKA were enriched in scorpion estrogen signaling pathway. Green color indicates up-regulated genes of adults.


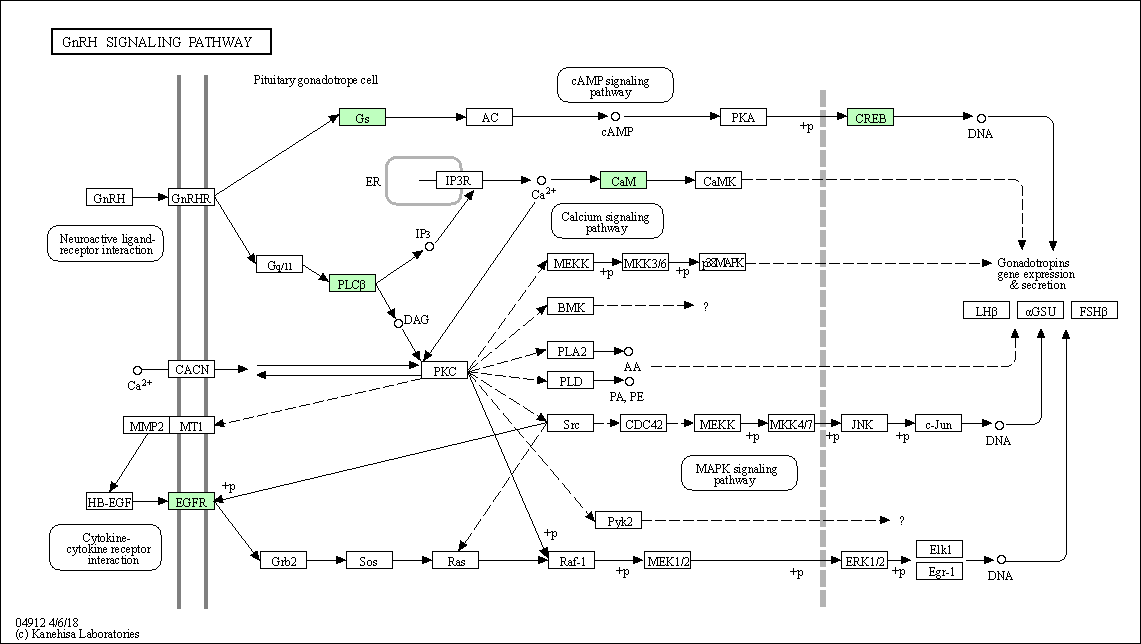


**Supplementary file 4.** GnRH signaling pathway. The up-regulated genes of Gs (GNAS), EGFR, CREB, PLCß and CaM (CALM), and the down-regulated gene of CACNA were enriched in scorpion GnRH signaling pathway. Green color indicates up-regulated genes of adults.


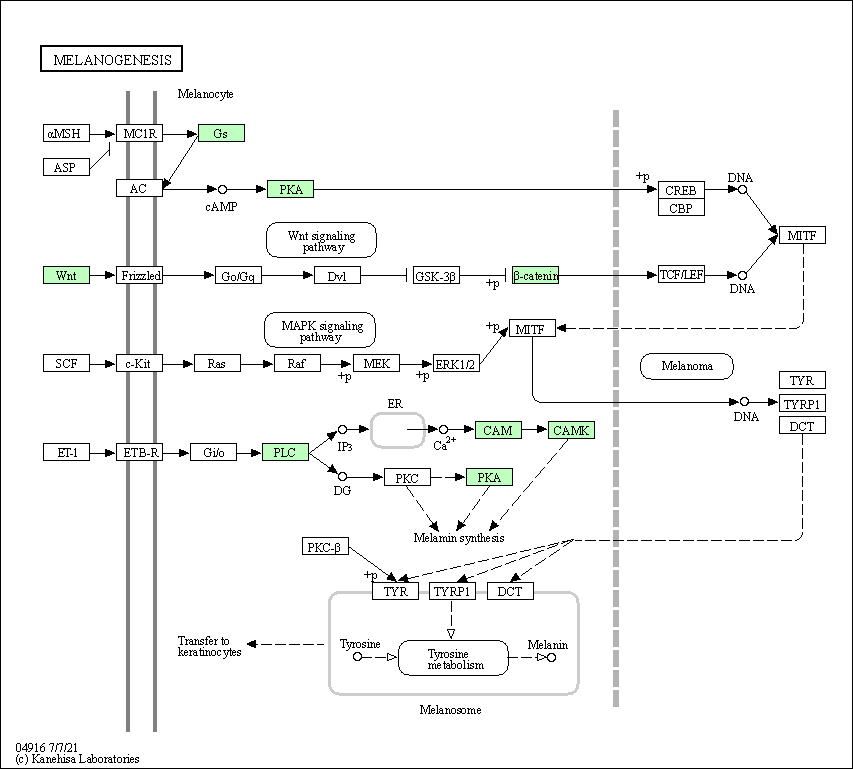


**Supplementary file 5.** Melanogenesis pathway. The up-regulated genes of GNAS, EGFR, CALM and PLCB, and the down-regulated genes of PKA and CAMK were enriched in Melanogenesis pathway.


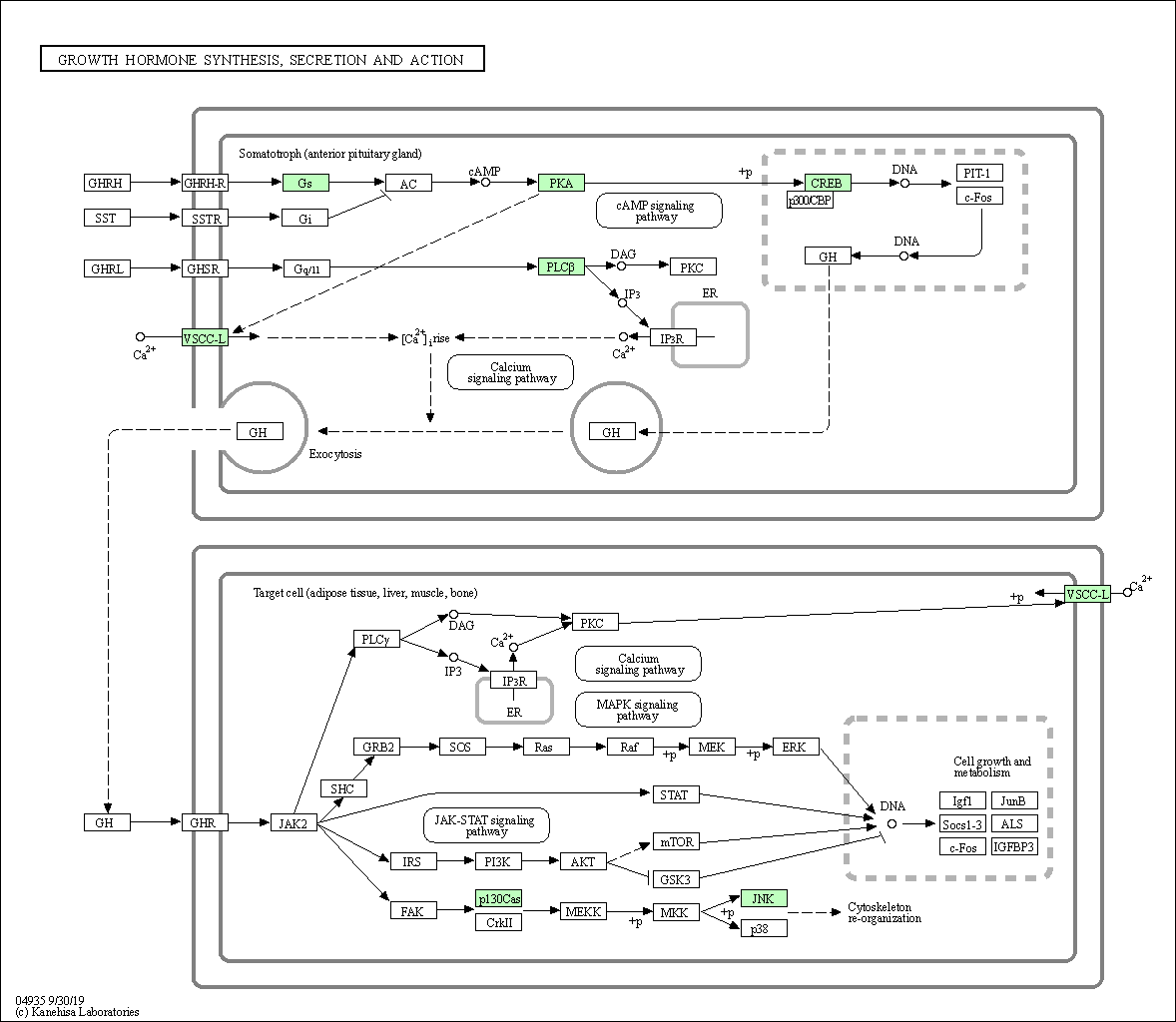


**Supplementary file 6.** Growth hormone synthesis, secretion and action pathway. The up-regulated genes of GNAS, CREB and PLCB, and the down-regulated genes of CACNA and PKA were enriched in scorpion growth hormone signaling pathway.

**Supplementary file 7.** Classification of up-regulated mRNAs into venom components**.**

**Supplementary file 8.** Classification of down-regulated mRNAs into venom components**.**
